# "I couldn't do this with opposition from my colleagues": A qualitative study of physicians' experiences as clinical tutors

**DOI:** 10.1186/1472-6920-11-79

**Published:** 2011-10-05

**Authors:** Bernhard von Below, Stig Rödjer, Mats Wahlqvist, Annika Billhult

**Affiliations:** 1 Department of Public Health and Community Medicine/Primary Health Care, The Sahlgrenska Academy at University of Gothenburg, PO Box 454, SE-405 30 Gothenburg, Sweden; 2 Floda Primary Health Care Center, Southern Älvsborg County, Rurik Holms väg, S-448 30 Floda, Sweden; 3 Section of Hematology, Department of Medicine, Sahlgrenska University Hospital, S-413 45 Gothenburg, Sweden; 4 Research and Development Unit, Primary Health Care, Southern Älvsborg County, Sven Eriksonsplatsen 4, plan 2, S-503 38 Borås, Sweden

## Abstract

**Background:**

Clinical contact in the early curriculum and workplace learning with active tutorship are important parts of modern medical education. In a previously published study, we found that medical students' tutors experienced a heavier workload, less reasonable demands and less encouragement, than students. The aim of this interview study was to further illuminate physicians' experiences as clinical tutors.

**Methods:**

Twelve tutors in the Early Professional Contact course were interviewed. In the explorative interviews, they were asked to reflect upon their experiences of working as tutors in this course. Systematic text condensation was used as the analysis method.

**Results:**

In the analysis, five main themes of physicians' experiences as clinical tutors in the medical education emerged: *(a) Pleasure and stimulation*. Informants appreciated tutorship and meeting both students and fellow tutors, *(b) Disappointment and stagnation*. Occasionally, tutors were frustrated and expressed negative feelings, *(c) Demands and duty*. Informants articulated an ambition to give students their best; a desire to provide better medical education but also a duty to meet demands of the course management, *(d) Impact of workplace relations*. Tutoring was made easier when the clinic's management provided active support and colleagues accepted students at the clinic, and *(e) Multitasking difficulties*. Combining several duties with those of a tutorship was often reported as difficult.

**Conclusions:**

It is important that tutors' tasks are given adequate time, support and preparation. Accordingly, it appears highly important to avoid multitasking and too heavy a workload among tutors in order to facilitate tutoring. A crucial factor is acceptance and active organizational support from the clinic's management. This implies that tutoring by workplace learning in medical education should play an integrated and accepted role in the healthcare system.

## Background

For more than twenty years, universities throughout the world have been introducing medical students to early patient contact and communication skills in a clinical context [1-4]. Research findings indicate that these courses are important in helping students early on in the curriculum to socially adjust to their profession and more confidently interact with patients while strengthening their biomedical learning [5,6]. Further, their transition to the clinical environment and clerkship is enhanced [7]. In early clinical courses, the tutor's role is important and includes activating students and encouraging them to learn from and reflect on encounters with doctors, patients and personnel in healthcare [8-10]. A learner-centered perspective, learning in context and workplace learning are central educational concepts used to facilitate student learning in early clinical experience courses [11,12]. These courses have often been evaluated from a student and outcome perspective. There are several articles about the important role of tutors in clinical education [12,13] and some regarding the essential qualities and skills required of a tutor [14]. Some papers report what tutors gain from teaching [2,15] but little is known about their views on working in these courses.

### Context

An "Early Professional Contact" (EPC) course was introduced in 2001 to the curriculum of the Sahlgrenska Academy, University of Gothenburg, Sweden. The course, which was compulsory, was set up as a longitudinal strand of student learning in clinical practice during the first two preclinical years, characterized by experience-based and small-group, workplace learning [6]. The aim of the course was to introduce students to clinical practice and the physician's professional role; to provide knowledge, skills, and inspiration for future work and to strengthen students' motivation for biomedical studies. Student-tutor continuity and a small-group learning method were key features of the course. The clinical tutor's main role was as a workplace instructor and facilitator to the student's learning process; to arrange access to learning experiences in practice and to provide space for reflexion, i.e. reflective tutoring [11]. A group of four students was scheduled to meet the same clinical EPC tutor in a process eight days/year. Each such practical training day involved a full day's clinical work together with the physician and staff. Reflective small group discussions were then facilitated by the tutor. Each student received a study guide; each tutor received an expanded teacher's version with the aim to both enhance tutoring and focus on to the aims of the course. In the guide, the course's learning goals were presented and aligned with tasks on each EPC practical training day at the clinic.

In the EPC course, both General Practitioners (GPs) and hospital specialists were engaged as tutors. Following the structure of the course, half were GPs and half were hospital specialists. The first EPC year was comprised of eight evenly distributed days on clinical attachments. During the second EPC year, students were relocated to new groups, from primary care to hospital attachments and vice versa meeting a new tutor for another eight days. Their clinical experiences took place at community-based clinics (Health centres) when the student group had a GP as a tutor, and at a hospital when the group had a specialist in a discipline other than Family Medicine as tutor. The course management recommended tutors to be relieved of routine clinical work during these EPC practical training days. Financing reward was provided by the university to the clinics and health centres to support this, the amount corresponding to the cost for substitutes for the tutors at the clinic.

Tutor meetings were held regularly once or twice per term to provide support and new information regarding the course, and to provide a forum for tutors to share experiences. These meetings were part of the task as a tutor but not mandatory. They were organized by the university, structured and held at the university. Tutors, as a bonus, were occasionally invited to lectures at the university on medical or humanistic subjects.

Tutors were recruited among interested colleagues with teaching experience. The tutor assignment was voluntary and without personal financial incentive. EPC tutors had to qualify by participating in a three-day, preparatory, tutor course. While leading this EPC course, the authors BB and SR noticed that some tutors resigned and they were curious as to the reasons for this.

In a previously published observational study, we found that tutors experienced a heavier workload, less reasonable demands and less encouragement, than students [6]. Clinical tutors are vital to modern medical education as role models and facilitators of students in workplace learning. A deeper understanding of the physician's perspective as a tutor might provide important clues for course development and facilitate tutorship. The aim of this study was to further illuminate physicians' experiences as clinical tutors.

## Methods

The research question aimed at a deeper understanding by illuminating tutors' experiences. In order to achieve this, a qualitative study based on explorative interviews with open-ended questions was conducted [16,17].

### Participants

In a letter, all 133 clinicians (both GPs and other specialists) with at least one year's tutoring experience of the new Early Professional Contact Course in Gothenburg were invited to participate in this study. Forty-seven responded and were interested (21 GPs and 26 other specialists) in participating; none formally rejected the invitation but 86 did not respond. In the invitation letter, they were instructed reply if interested in participating in the study. A purposeful sample was used, aiming toward diversity of age, sex, subspecialty and location. Twelve physicians were interviewed, six GPs and six from other specialties (two internists, one geriatrician, infectionist, gynecologist and orthopedist). Six men and six women participated, aged 39 to 61 years. Half of them worked geographically close to the university hospital of Gothenburg, and the other half on the outskirts of the city or in smaller cities.

### Data collection

Data was collected by explorative interviews [16] with open-ended questions starting with the opening phrase:"Tell me about your experiences of work as a tutor in the EPC course". The aim was to focus on experiences of the physicians regarding their work as tutors, hence deepening follow-up questions were included: "Please tell me more", "How did that feel?". Interviews lasted 45 to 75 minutes and were conducted at the informants' workplaces between September 2006 and June 2007.

As a GP, the first author (BB) interviewed the hospital tutors and as a hospital-employed physician, the co-author SR interviewed the GPs in order to avoid disturbances from intradisciplinary pre-understanding. All interviews were recorded digitally and transcribed verbatim.

### Ethics

Ethical approval was obtained from the Regional Ethical Review Board at the University of Gothenburg. No informants reported negative experiences during or after the interview. During transcription, names were removed to ensure confidentiality.

### Analysis

Systematic Text Condensation (STC) as described by Malterud [17,18] was used as a method of analysis. STC is especially useful in a descriptive and transverse analysis of phenomena based on the experiences of many informants. The method is inspired by Giorgi [19], and then modified by Malterud [17,18]. Analysis proceeded through four stages:

(a) reading the entire material for an overall impression, bracketing preconceptions; (b) identifying units of meaning, representing different aspects of the tutors' experiences and coding for these; (c) condensing and abstracting the meaning within each of the coded groups; and (d) summarizing the contents of each code group to generalized descriptions and concepts reflecting the most important experiences reported by the informants. Using this method, categories were developed from the empirical data. The purpose of citations in the text is illustrative. The authors adopted a learner-centered perspective in tutoring and adult education as the theoretical frame of reference [8-10].

## Results

In the analysis, five main groups of experiences of physicians as clinical tutors in the medical education emerged; (a) Pleasure and stimulation, (b) Disappointment and stagnation, (c) Demands and duty, (d) Impact of workplace relations, and (e) Multitasking difficulties.

### Pleasure and stimulation

Informants reported pleasure and stimulation from tutoring, both from meeting students and tutor colleagues. It also gave them a break from daily clinical work.

#### Students brought pleasure

Tutoring was pleasant in several ways. Informants felt appreciated and confirmed. Meeting young students was considered positive, as was the possibility to influence and form future physicians. Tutors were stimulated by the students' many questions and their positive feedback, as illustrated by this tutor:

*The students' enthusiasm and the chance to influence future physicians, to do something good for those that form them into good (physicians)...was a pleasure*. //

*It's fun to meet with them. You get so much in return; you become their idol, a role model*, *really. Everything you say they consider pearls of wisdom (laughs), which is a bit of a kick. ---To be able to provide these...precious moments are very, very pleasant*.

#### Inspiration by collegial exchange

Fellowship with other tutors was inspiring. Meetings and lectures presented a possibility for sharing experiences with tutor colleagues. Preparatory tutor education was found interesting and stimulating, providing new pedagogical knowledge and enhancing consultation skills. Informants expressed inspiration by meeting other colleagues and experienced collaboration between disciplines, such as family medicine and those of the hospital world, illustrated this way:

*The fellowship I've experienced with other tutors has been tremendously stimulating, and in particular, getting to meet other physicians, since you don't get much chance as a clinician*.

The task as a tutor also meant a break from daily clinical work, thus providing stimulation, as this 52-year-old GP expressed:

*It's something that breaks up the workday. I love my workday filled with patients, that's perfectly all right, but it's nice when...something else to look forward to breaks things up a bit*.

### Disappointment and stagnation

Occasionally, tutors expressed negative feelings such as disappointment or stagnation.

#### Disappointment

Informants reported disappointment due to patients refusing to take part in agreed student examinations or when the course management did not ask them to continue as tutors. Unprepared students, disregarding previous agreements of preparation, also generated feelings of disappointment, as illustrated by a GP tutor:

*The last few years, they hadn't read what we had agreed upon --- that sort of thing, gets you more and more tired of it, --- since I had read all the articles --- Went to the library and borrowed books and so on and then they [the students] had not read them*.

#### Stagnation by time

After being a tutor for several years, a sense of routine and stagnation might appear causing enthusiasm to dwindle, as for this 61-year-old tutor at a hospital said:

You can imagine after 3,4,5 years, perhaps it's time for a break to get a little... Everything becomes routine in some way

### Demands and duty

#### Giving your very best to improve education

Being a tutor was expressed as a commitment but also as a demand. Informants had accepted the task as tutors and felt responsible for fulfilling this task satisfactorily, here expressed by this GP:

*It is, in any case, a task you take on, that you want make something good out of. And I think it is important for it to go well. So I probably felt a responsibility for it*.

The informants expressed an ambition to give students their best, a desire to make a valuable contribution to their education. The tutors often referred to their own medical studies. Their ambition was to provide a better medical education, to give the students something, which the tutors themselves did not have as medical students. Tutors expressed an ambition to be role models. Both ambitions are illustrated here by these tutors:

*So, I probably missed this myself in my medical schooling....I'm really hoping for a little better education for them or to have clinical contacts from the start*.

*Yes, I think it is important that there are doctors that retain a wider perspective both in their role and profession*. *And yes, we have some sort of responsibility for the coming generation..*.

Preparing took time and energy. Tutors worked hard to find meaningful activities for the students, to find patients and colleagues who could take part in these activities. It was exerting to form the day to the students' satisfaction, as this internist at a university hospital said:

*Needed to find patients, find colleagues who could participate, find a place, and find time for myself to participate --- Yes, I thought this was a disadvantage of the project*.

Informants also felt a duty to enhance the recruitment of tutors and an obligation to meet demands from the course management such as using recommended study guidelines. Among GP informants, there was also a wish to recruit forthcoming colleagues and point out the key features and values of family medicine. A GP working in a small town expressed the following:

*We can give them a good education. We can show them, I believe, at an early stage, that a GP is a physician, a specialist respected by surgeons and internists and so on. Because we are so capable, I think*.

#### Difficult situations with students

Tutors experienced multiple demands arising from interactions with students, situations arose pressuring tutors to handle difficulties they were not prepared for. As one tutor said:

*And then I had, what I felt was a difficult conflict with one of the students who was, who said many odd things that we all reacted very strongly to but couldn't.... I couldn't go further with it; I didn't know how to deal with what he had said*.

Feedback to students was considered difficult, sometimes creating a feeling of inadequacy. One tutor expressed this in the following way:

*The ability to provide meaningful feedback to the students, we are poorly trained for that, too. That is a valuable moment in tutorship*.

#### Tired but satisfied

After a day supervising students at the clinic, informants were tired, yet satisfied. A feeling of exhaustion was, however, combined with satisfaction. Their ambition for the day in clinical practice to turn out as planned and be helpful to student learning took much energy. For some informants, this was almost overwhelming, as expressed by this GP:

*I was tired, completely exhausted, you know. Satisfied but still a demanding effort. Tired, yes, oh yes. You're really tired after such a day*.

### Impact of workplace relations

Colleagues and staff accepted meeting students when well planned and even more so when informants had the support of the clinic's management in their role as tutors.

#### Workplace support

Informants reported tutoring easier if colleagues accepted students coming to the clinic, although this sometimes meant additional work for them, which they sometimes reported as irritating. If their time was required they demanded to be informed well in advance. Further, only a small number of students at a time were accepted. Young colleagues often showed great interest in meeting and teaching students. Other staff members were reported as mostly positive toward meeting students and were stimulated by the possibility of showing them their tasks and functions at the workplace, as this tutor said:

Most healthcare personnel enjoy showing what they do, if they don't have too many students

#### The importance of planning and backup

When planning was inadequate, colleagues and staff members at the clinic were sometimes negative to participation in student tutoring, especially when not informed in advance or if there were many students. Informants reported sometimes having an unpleasant feeling when burdening colleagues and staff by asking them to participate in meeting students. Tutors also expressed a feeling of loneliness, lacking support and understanding, caused by the additional work that supervising students often entailed. They felt inconvenienced, asking for help from colleagues, as illustrated here by a 44-year old GP:

*If I sighed a bit at the end of the day and said: now the students have gone home, now I have to make those 10 phone calls that have been waiting, so my colleagues thought 'you've got yourself to blame'. I didn't mean they should do it for me, but it was a bit tiresome hearing that attitude*.

#### Clinic's management support enhanced tutorship

When the clinic's management actively supported student placement at the clinic, there was a better acceptance of tutorship at the workplace, among colleagues and staff. Then the roles of clinician and tutor were more easily combined. Tutors felt freer in taking time from patient-related work for preparing student activities and students were also more easily accepted at the clinic, as this tutor at a smaller hospital expressed:

*The task is sanctioned by my boss and...... Yes, I think that's very important, then...first then do you feel that the job is respected and taken seriously and you can be devoted to it..... I couldn't do this with the opposition from my colleagues...if they think that, yes, there he goes goofing off with those students. But that is not the way I experience it at all*.

### Multitasking difficulties

Combining several duties as a physician with that as a tutor was often reported as difficult.

#### A tutor - still a clinician

Several informants expressed difficulties combining the tasks of tutor and clinician. Often, a conflict arose between either taking care of students or of patients. When the students left at the end of the day, much patient-related work remained. Even if the tutor was scheduled to handle students, he or she was seldom regarded as free of clinical duties. When there was shortage of colleagues at the workplace, the day became hectic. Tutors often found it difficult to find time to prepare student visits instead of seeing patients. Meeting patients was of the highest priority, here illustrated by a tutor at a university hospital:

*I believe the healthcare personnel know we have other things to do but they probably think, and we as well, that in some ways patients are of the first priority. --- There's no one else that can do this job. My other workdays then get longer*.

#### Tutoring as an additional and optional activity

The informants also expressed being involved in, or were encouraged to get involved in, other tasks and functions beside those of tutorship and clinical duties. They felt they had too many tasks or too little time. When prioritising among these tasks, supervising students was not compulsory. The assignment as a tutor was regarded as an additional and optional activity. Refraining from the task of tutor could then be experienced as a relief, as this tutor said:

It probably has to do with how many other projects you're involved in. So now that I've become involved in union work since last year, it's definitely too much. And then being a tutor is clearly something that can be eliminated

## Discussion

The analysis of the study identified five main themes of experiences of physicians as clinical tutors in the medical education: Pleasure and stimulation, Disappointment and stagnation, Demands and duty, Impact of workplace relations, and Multitasking difficulties.

### Methodological discussion

Limitations in this study concern credibility. Two of the authors (BB, SR) held positions as course leaders in the EPC course, from which informants were recruited. Our perspective in this paper is that of the tutors and not the students. In the course leadership, BB as a general practitioner was responsible for the GP tutors and SR, as an internist, for the tutors from the hospitals. Together with several colleagues, we discussed the risk of disturbance from preconceptions. Bracketing preconceptions is important in study designs with a phenomenological approach [19]. Accordingly, the authors did not interview tutors with whom they had worked or addressed as course leaders. This was made possible by means of "crosswise" interviewing; BB interviewed hospital tutors and SR interviewed GP tutors. The authors discussed this thoroughly before the interviews to reduce the impact of pre-understanding.

On the other hand, the interviewer's knowledge of the interviewees and their environment may have enhanced communication [20,21]. In order to increase trustworthiness, the analysis was conducted together with the co-authors SR, MW and AB. All authors read the interviews and participated in the analysis. Our theoretical frame of reference is presented above (see Method section).

Among the forty-seven tutors willing to participate in the study, we chose a purposeful sample with diversity of age, sex, subspecialty and geographical position to increase validity. As all tutors, informants were engaged as tutors at the EPC course on a voluntary basis. This, as well as the fact that participation in the study was voluntary, may have influenced results, as these physicians might have been especially committed and ambitious about tutoring. We have not been able to study the non-responding clinicians, but they may have been busier or less interested in tutoring. However, the sample was varied, the material obtained was rich and many aspects of tutors' experiences emerged in the interviews. Thus, we regard it possible to apply our findings concerning tutors' experiences beyond the context of this study, although the fact that tutorship for this EPC course was voluntary might reduce transferability to a context where this is not the case.

### Discussion of results

An important experience was the feeling of *pleasure and stimulation*. Most informants started their interviews by expressing this feeling. It is obvious, that tutoring is appreciated. Meeting students is stimulating *per se*, both by meeting young people but also due to the appreciation and positive feedback from students. Tutors seem to gain self-confidence by meeting students. Earlier research concerning GP tutors [2,15] supports these findings. However, in our study the tutor group was broader including GP tutors as well as tutors from other disciplines.

At tutor meetings and preparatory tutor education, there was time and opportunity to discuss and reflect together with colleagues in a way seldom possible in everyday clinical work due to stress and lack of time [22]. Tutorship was thus stimulating by meeting both students and colleagues.

The informants initially reported these positive feelings. However, using open interviewing may also enhance the appearance of other ambiguous experiences [23]. Here, feelings of demands and duty were expressed by the informants, as was frustration towards multitasking and lack of time. These findings confirm and give us an opportunity to better understand the results of a previous paper [6], where tutors experienced a greater workload, less reasonable demands and less support, than students.

These *demands *seemed to be mainly internal, coming from a commitment to the task and a desire to give today's students a better education than what the tutors felt they once were given. Curriculum overload and cramming in traditional forms of medical education have caused negative experiences among many students in the past [12]. The ambition of our tutors might have been a reaction to such negative self-experiences. It is well known, at least among GPs, that deficits in their personal medical education might increase motivation for tutoring [24]. Of course, this is positive regarding the educational result in a shorter perspective. However, as feelings of extreme tiredness and even exhaustion from too heavy a workload after days in the clinic with students are expressed, it might be negative in the long run. The lack of a reasonable workload might increase the risk for tutors quitting. Informants also expressed other internal demands, such as the duty of recruiting new colleagues to their specialty and new tutors to the course. This view was expressed particularly by the GPs. Tutors wanted also to serve as good role models for the students. This leads to a picture of an ambitious, active physician committed to the task of tutor.

It is noteworthy to mention that tutorship at this time was optional in this course, so there might have been an active selection by these committed tutors.

Some of the demands can be described as external and a result of intentions by the EPC course management when implementing this new course. The form of tutorship in this course was new for the physicians (a more active tutorship, small-group tutoring, recommendations in a study book specific for the course, and engagement as tutor for the student group lasting as long as two terms) [6].

Hence, it is important that tutors are adequately educated and that aims, course content and tutor tasks are well defined and clearly communicated. The importance of being well prepared, when beginning as a tutor, is well known [25] and our findings support this. Tutors need to be prepared in small group tutoring when a course, such as this EPC course, is based on workplace learning in small groups.

A specific situation eliciting feelings of insufficiency was *giving feedback *to students. The literature on feedback in medical education including workplace learning is growing rapidly. Feedback can have a powerful effect on learning but demands preparation and time [26,27]. Our results suggest that learning how to provide feedback is an important part of a tutor's competence.

Obviously, physicians' *multitasking *is a critical factor regarding their willingness to accept tutorship. A physician's day is normally filled with clinical work but there are other activities that may attract or engage physicians, for example research or quality-developing activities. Multitasking *per se *might have a negative impact on cognition, health and professional behavior [28,29] causing a lack of time for each task.

Informants expressed frustration towards lack of time. They particularly emphasized the conflict between their normal clinical activities and tutorship. It is obvious that the recommendation from the EPC course management that tutors should be relieved of routine clinical work during these EPC practical training days and giving financial support to the clinic to enable this [6] was not enough.

It is interesting to note that tutorship is regarded as a task, easy to reject when pressure from multitasking grows.

Consequently, it is necessary for tutors to have scheduled time for tutorship and reduce their clinical activities and duties. Involvement in medical education in terms of clinical tutorship must be recognized as an important part of a physician's work.

The *impact of workplace relations *are also influenced by the common view that a tutor's first responsibility is to clinical duties, even when working as a tutor.

Staff nurses in particular voice this opinion, according to informants, but support from colleagues also appears to be lacking. Much clinical work remains after students have left. However, when planning was adequate and colleagues and staff were prepared in advance, they were often positive and willing to accept students' presence at the clinic. This shows the importance of good planning both from course leadership at the university as well as from tutors.

Our results show, that *clinical management support *is essential and enhances tutorship. When the tutor has this support and acceptance it is much easier to schedule time for tutoring and inform colleagues and staff about the importance of the task as a tutor and its time-consuming consequences. It also becomes easier to gain acceptance for tutors' withdrawal from clinical duties during the time when students visit the clinic thus reducing multitasking.

## Conclusions

It is important that tutors' tasks are given adequate time, support and preparation. Accordingly, it seems highly important to avoid multitasking and too heavy a workload among tutors in order to facilitate tutoring. A crucial factor is acceptance and active organizational support from the clinic's management. This implies that tutoring by workplace learning in medical education should play an integrated and accepted role in the healthcare system.

## Competing interests

The authors declare that they have no competing interests.

## Authors' contributions

BB, SR and AB have made substantial contributions to the conception, design, acquisition and analysis of data and have been involved in drafting the manuscript. AB has also been involved in critically revising the manuscript. MW has made substantial contributions to the analysis and interpretation of the data, and was involved in revising the manuscript critically for important intellectual content. All authors read and approved the final manuscript.

## Pre-publication history

The pre-publication history for this paper can be accessed here:

http://www.biomedcentral.com/1472-6920/11/79/prepub
